# A Real-Time Semantic Map Production System for Indoor Robot Navigation

**DOI:** 10.3390/s24206691

**Published:** 2024-10-17

**Authors:** Raghad Alqobali, Reem Alnasser, Asrar Rashidi, Maha Alshmrani, Tareq Alhmiedat

**Affiliations:** 1 Saudi Data and AI Authority, Riyadh 12382, Saudi Arabia; ralqabli@sdaia.gov.sa; 2 Information Technology Department, Faculty of Computers and Information Technology, University of Tabuk, Tabuk 71491, Saudi Arabia; 431010436@stu.ut.edu.sa (R.A.); 431007275@stu.ut.edu.sa (A.R.); 431000057@stu.ut.edu.sa (M.A.); 3 Artificial Intelligence and Sensing Technologies (AIST) Research Center, University of Tabuk, Tabuk 71491, Saudi Arabia

**Keywords:** robot navigation, semantic maps, path planning, robot vision

## Abstract

Although grid maps help mobile robots navigate in indoor environments, some lack semantic information that would allow the robot to perform advanced autonomous tasks. In this paper, a semantic map production system is proposed to facilitate indoor mobile robot navigation tasks. The developed system is based on the employment of LiDAR technology and a vision-based system to obtain a semantic map with rich information, and it has been validated using the robot operating system (ROS) and you only look once (YOLO) v3 object detection model in simulation experiments conducted in indoor environments, adopting low-cost, -size, and -memory computers for increased accessibility. The obtained results are efficient in terms of object recognition accuracy, object localization error, and semantic map production precision, with an average map construction accuracy of 78.86%.

## 1. Introduction

The term “robot navigation” refers to autonomous robot systems’ ability localize mobile robots and plan a route between points of interest. Robot navigation is an essential task for a wide range of applications, including military, industrial, agricultural, home automation, and social tasks [1,2]. The robot navigation involves a set of tasks. These include scanning the environment of interest, constructing a geometry map, performing path planning, and exploring the shortest paths between different points. In general, the success of the robot navigation system depends on the accuracy and detail of its map [3,4].

To create these maps, there are two commonly used maps in robotics: geometric and topological [5]. Geometric maps employ geometric features or grids to present the layout of the environment, whereas topological maps represent the environment using graphs; these represent places as vertices in the graph and their interrelation as links.

One of the current systems, the simultaneous localization and mapping (SLAM) system, allows the robot to build a geometric map of its surroundings and locate itself within the environment. However, geometric maps are inefficient in complex indoor environments. Therefore, there is a high demand to build a rich map with semantic labels through the employment of vision-based systems.

Typically, the construction of a semantic map requires the employment of a visual system in order to perceive and recognize objects in an area of interest. Therefore, visual perception is one of the key capabilities of smart robotic systems, enabling them to adapt in their environment. The concept of semantic information represents classes of objects in the area of interest in order to allow the mobile robot to understand its surroundings more completely than if it used only a geometric map. This increased detail allows a robot with semantic data to perform better in human–robot interaction, path planning, and indoor navigation.

Whenever the semantic information represents classes of objects, a mobile robot can understand its surroundings better. Therefore, it is important for the mobile robot to perceive and understand the objects inside its environment; when it encounters an obstacle, it needs to act accordingly. Semantic navigation allows for this, and it offers the following significant benefits to the area of mobile robot navigation [5]:

1. It is more human-friendly. The robot platform understands the environment in the same way a human understands it.
2. It is autonomous. Through semantic navigation, a robot can perform independent action(s) as long as it understands its environment.
3. It is efficient. The robot does not need to explore the entire environment to decide its route. Instead, it can choose its path based on the fastest or shortest route.
4. It is robust. The robot platform can recover missing navigation information.

Because of these benefits, this paper focuses on semantic map representation for the purpose of indoor robot navigation. The work presented in this paper aims to develop an efficient map production system that is able to generate a semantic map for indoor environments that will improve the efficiency of path planning tasks. Therefore, the main contributions of this paper are as follows:

1. It discusses recently developed semantic navigation systems for indoor robot environments.
2. It designs an efficient semantic map production system for indoor robot navigation.
3. It assesses the proposed system’s efficiency using the robot operating system (ROS) development environment and through the employment of a set of reliable validation metrics.

The remainder of this paper is organized as follows: Section 2 discusses semantic navigation for robot systems; the proposed system design is presented in Section 3. The experimental testbeds and results are discussed in Section 4. In Section 5, the results are discussed and compared with other recently developed semantic navigation systems for indoor environments. Finally, Section 6 concludes the paper and discusses future work.

## 2. Related Works

In general, the maps produced by robot exploration fall into the following two categories: metric maps that represent the geometric information about the environment and topological maps that present distinctive points. The metric and topological maps are sufficient to provide the robot with a basic structure to navigate its environment. However, the development of semantic maps could allow a robot to understand its environment in the same way a human does, as these maps not only offer geometric information about the environment but also offer high-level semantic information that can be used to allow the robot to perform several tasks in automated manners. This would benefit the field of robot navigation.

**Figure 1:**
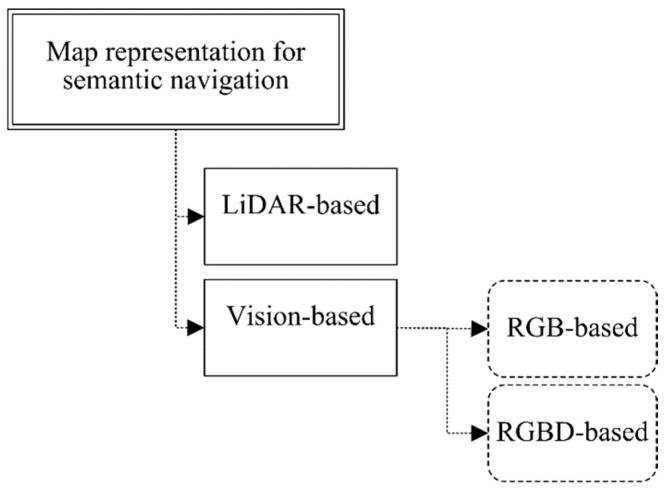
Classification of map representation approaches for robot semantic navigation.

Existing robot semantic navigation systems have been discussed and analyzed in a recent study [6]. However, this paper focuses on map representation systems using vision-based subsystems for the purpose of constructing a semantic map. To achieve this, the existing robot map representation systems are categorized according to the perception systems that they employ, either LiDAR- or vision-based approaches, as presented in Figure 1.

LiDAR-based systems process and analyze the signals reflected from the LiDAR unit for the purpose of classifying the area of interest based on a pretrained model. Several research works have focused on semantic mapping using LiDAR technology due to its simple implementation and lower cost.

For instance, the work presented in [7] utilized a semantic exploration system for indoor robots; the developed system was able to classify four different indoor places (door, room, hall, and corridor) with a classification accuracy greater than 95%. On the other hand, the authors of [8] developed an obstacle classification system through the employment of two different LiDAR units, whereas the work presented in [9] produced a classification system for persons in complex environments. Moreover, in [10], the authors developed a semantic classification system in order to categorize three different types of buildings, non-residential, single-family, and multiple-family buildings, with a classification accuracy of >70%.

As presented above, LiDAR-based navigation systems are efficient in terms of overhead requirements, including memory size and processor speed. However, the LiDAR-based systems [7,8,9,10] offer limited classification capabilities (between one and four classes) due to the nature of the data collected from the LiDAR unit. Vision-based systems are an alternative to LiDAR. These involve employing RGB or RGB-D vision systems in order to better explore the navigation area. For instance, the work presented in [11] employed semantic segmentation and detection through the adoption of a deep network model to learn the navigation policy. In [12], the authors proposed a framework for the purpose of building an improved metric representation of the environment using semantic information, in which the system’s output was a map that included semantic object classes and their locations.

In [13], the authors developed a real-time solution for the problem of detecting and recognizing hallway scenes from RGB images, in which a multi-scale fully convolutional network (FCN) was employed to predict the scene. The work presented in [14] included the design of a semantic simultaneous localization and mapping approach for rescue robots; the developed system was able to generate a geometric map with semantic information. The authors of [15] presented a semantic segmentation network to develop an indoor robot navigation system, in which the developed CNN-based system was successfully able to classify the environment type.

The work presented in [16] focused on semantic knowledge and discussed how it can be used for robot task planning. They conducted several experiments that demonstrated the effectiveness of their proposed solution in indoor robot navigation in domestic environments. The authors of [17] proposed an efficient visual SLAM approach using RGB and depth images to enhance hospital operation and minimize the risk of doctor–patient cross-infection. A vision-based navigation system was proposed in [18] to detect roads in outdoor environments using deep convolutional neural networks.

In [19], the authors discussed the process of building semantic maps and how to interactively label entities and use this information to enable context-aware navigation behaviors in indoor environments. The work presented in [20] involved the design and development of an object semantic grid mapping approach through the employment of a 2D LiDAR and RGB-D camera to obtain semantic information for robot navigation. The LiDAR unit was employed to obtain an occupied grid map and decide the robot’s trajectory. In the second stage of this study, the authors employed object detection to obtain objects’ semantics color images using joint interpolation.

A novel semantic map construction system that combines geometric information and vision data is presented in [21]. The conducted experimental results demonstrated the effectiveness of the proposed semantic map production approach. On the other hand, the work presented in [22] proposed an interactive approach for constructing a grid-semantic map for the navigation of service robots in indoor environments. The developed system has been validated using the ROS framework through the implementation of a number of experiments that were carried out in simulated and real environments.

As discussed above, vision-based systems have been employed for the purpose of constructing a rich semantic map. However, the integration of both LiDAR- and vision-based units produces an efficient and rich semantic map that includes geometry details and the objects in the navigation environment.

## 3. Semantic Map Representation Approach

**Figure 2:**
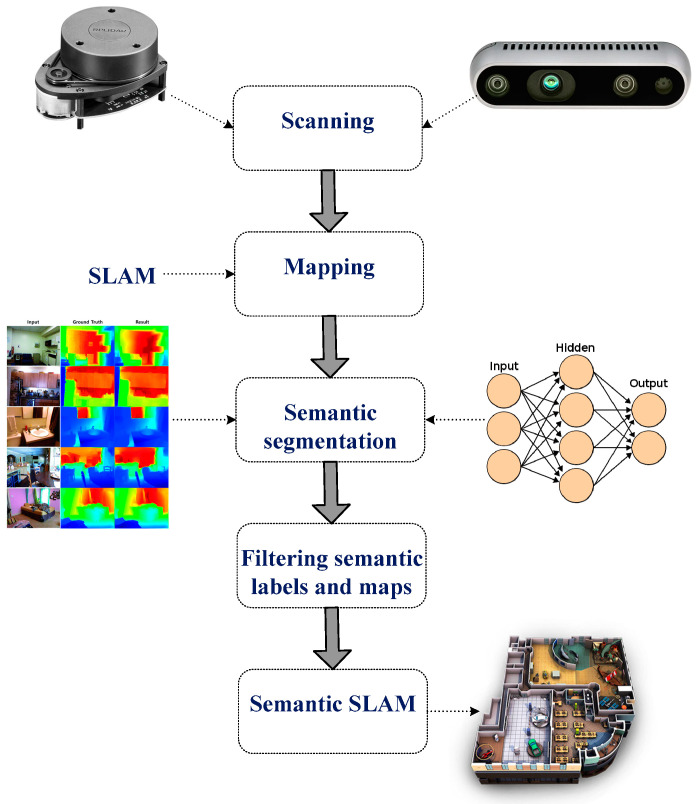
Main stages of the developed semantic map production system.

For efficient robot navigation, an accurate map needs to be obtained using vision and LiDAR sensing units. This section discusses the system design for the proposed semantic map production system, which consists of five main phases, as presented in Figure 2.

The first phase involves scanning the environment of interest using a LiDAR unit (RPLidar A1M8 device) and RGB-D camera (OAK-D Pro—Luxonis). The former collects the geometry information of the area, whereas the latter collects relevant information about objects in the area of interest for future object detection and classification. In the second phase, the geometry map is established using the data obtained from the LiDAR unit, and possible paths between points are determined and estimated.

The third phase involves semantic segmentation, which includes object detection and recognition tasks. The robot system needs to recognize objects of interest in the navigation environment through the adoption of an efficient object detection algorithm; this allows it to construct an efficient and rich semantic map. To achieve this task, the you only look once (YOLO) v3 model has been employed, which has been trained using two vision datasets, the Pascal dataset (20 different object classes) and the COCO dataset (80 different object classes), and Table 1 presents the object classes for both datasets.

Moreover, Table 2 presents a summary of both datasets (COCO and Pascal) that includes the total number of records, the number of classes, and the total memory size. The COCO dataset targets indoor applications, as it consists of objects that may exist in domestic applications. On the other hand, the Pascal dataset aims to be deployed with outdoor object detection approaches. The combination of two datasets allows the developed system to be employed in indoor and outdoor environments for the purpose of constructing an efficient semantic map.

YOLO is an efficient object detection model that has been developed widely in robot navigation systems [23,24,25]. According to the recent experimental study presented in [26] that has implemented and validated several vision detection models for robot navigation applications, each detection model has been validated using several parameters, including object detection rate, object detection accuracy, and processing time. The obtained results proved that the YOLO detection model achieved better classification accuracy than other detection models.

In the fourth phase, semantic labels are filtered. The detected objects are labeled and located in the navigation environment based on the objects detected from the previous stage, in which this task is accomplished based on the employment of the RGB-D camera. Table 3 presents the structure of the constructed semantic table that consists of an identification number for each detected object, along with the 2D position coordinates (x-cord and y-cord).

The semantic map is generated in the fifth phase, which involves building a semantic SLAM map that includes both the geometry information and the detected objects and their corresponding locations in the navigation area in a way that the robot platform can understand them. The objects and their corresponding locations are recorded in a semantic table (presented in Table 3) located in the robot’s main memory.

**Figure 3:**
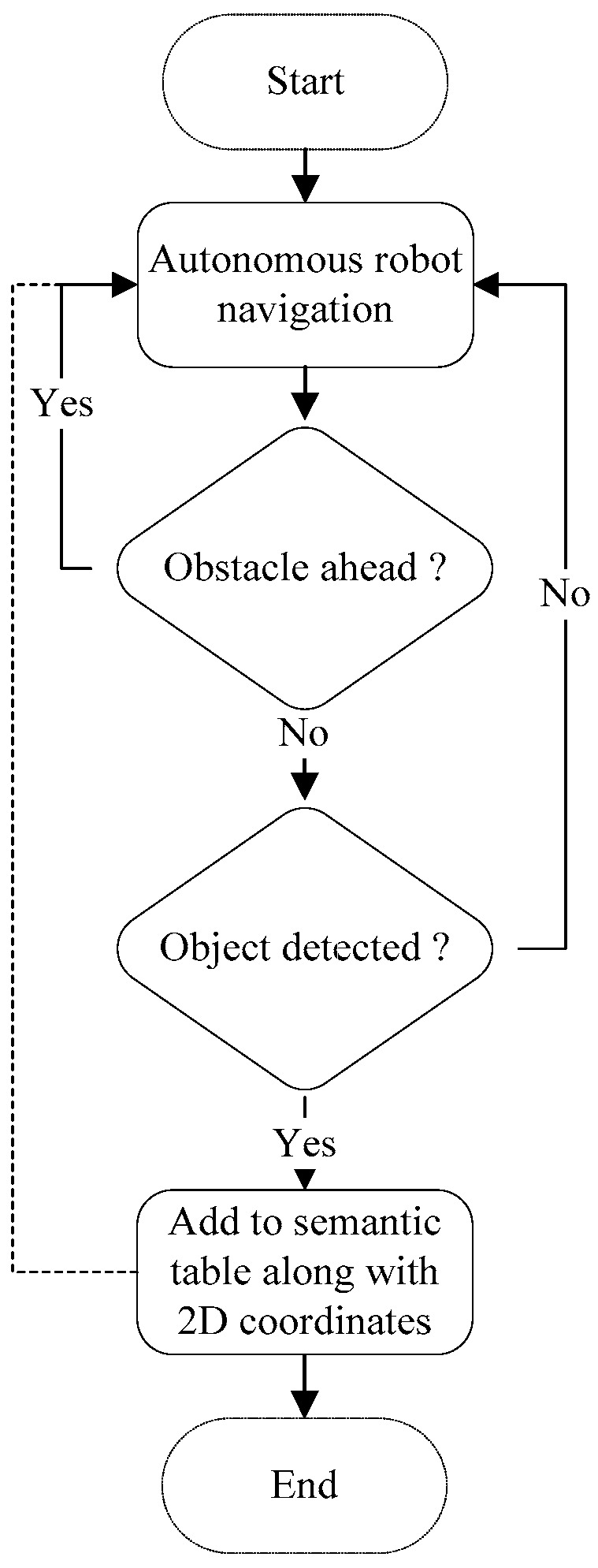
Flowchart for the semantic map production function.

The map production function is presented below in Algorithm 1, in which the main attributes and methods are presented. The flowchart of the semantic map production function is presented in Figure 3, in which the robot navigates the area of interest and avoids obstacles that may exist. Then, the robot detects and recognizes the objects using the pretrained YOLO model, and finally, the detected objects are recorded in a semantic table.
**Algorithm 1:** Semantic Map Production01: let *A_x,y_* is the 2D navigation area with the dimensions of *x* as width and *y* as height 02: let *mr* is the mobile robot in the navigation environment 03: let *mr(x,y)* is the current 2D location of the mobile robot in the navigation environment 04: let *mr_maxX_* is the maximum reached point by *mr* at the *x*-axis 05: let *mr_maxY_* is the maximum reached point by *mr* at the *y*-axis 06: let *yolo_CP_* is the trained model on two datasets: COCO and Pascal 07: let *depth_to_object_k_* is the depth distance to the detected object *k* 08: let *obs_dist* is the distance in centimeter (cm) to the heading object 09: let *navigate_fun* is the navigation function in the area of interest 10: let *sem_table* is the semantic table that includes a list of objects along with 2D coordinates 11: while (*mr_maxX_ < x && mr_maxY_ < y*): 12:         while *obs_dist* > 100: 13:                 if (*object_detected*(*yolo_CP_*, *depth_to_object*)): 14:                                         *sem_table*(*object_detected, mr(x,y)*) // add the new detected object along with its 2D coordinates 15:                 else: *navigate_fun* 16: end

## 4. Experimental Results

This section discusses the hardware and software requirements required to implement the proposed map representation system. It also discusses the developed system in context with the robot operating system (ROS) development environment. Finally, the results obtained from simulation experiments conducted in an indoor environment are discussed and analyzed.

### 4.1. Development Environment

**Figure 4:**
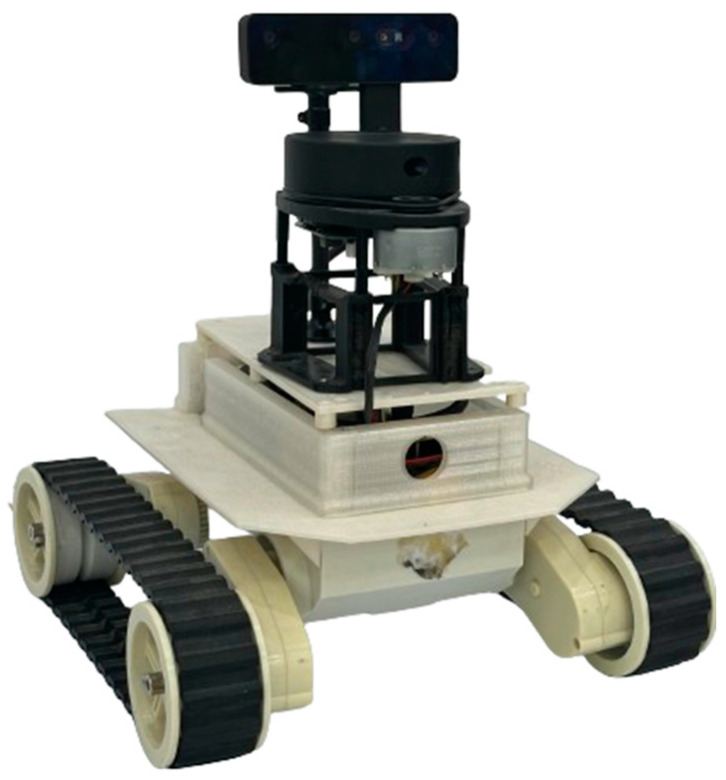
Simulated two-wheel-drive robot platform.

**Figure 5:**
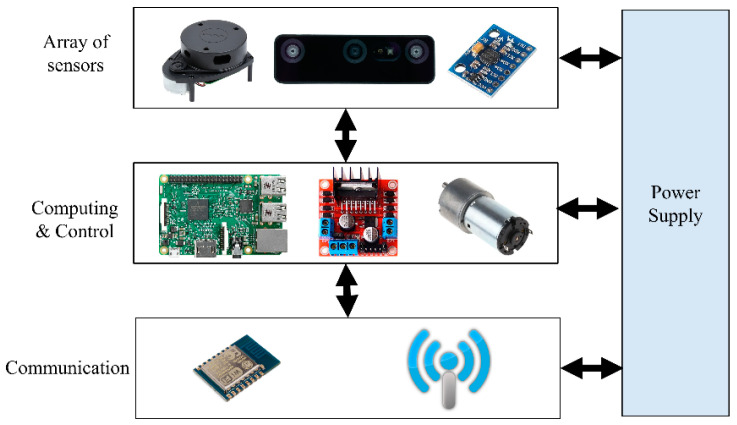
Architecture of the simulated robot platform.

A real robot platform has been simulated into ROS in order to build a reliable semantic navigation system. The customized robot platform is a two-wheel-drive robot based on a rover platform, which is depicted in Figure 4. A Raspberry Pi 4 (4-GB RAM) computer was integrated to process the data received from onboard sensors (LiDAR and RGB-D units) and construct a semantic map. The robot architecture is presented in Figure 5, and Table 4 presents the developed robot’s specifications.

The robot platform was able to capture RGB-D images using an OAK-D pro camera unit and then process the captured images on the Raspberry Pi computer for the purpose of minimizing the communication overhead with a remote server. The average frame per second (FPS) ratio was set to four in order to minimize the overhead processing on the Raspberry Pi computer. Through the experimental studies, four FPS was considered reasonable, as the robot speed was set to 10 m per minute.

On the other hand, the LiDAR unit was employed to measure the distances between the robot platform and heading objects or walls for the purpose of constructing the geometry information of the area of interest. In the experimental studies, the LiDAR frame rate (LFR) was set to 20 frames per second, and this value was practical with the adopted robot platform and the navigation scenario.

**Figure 6:**
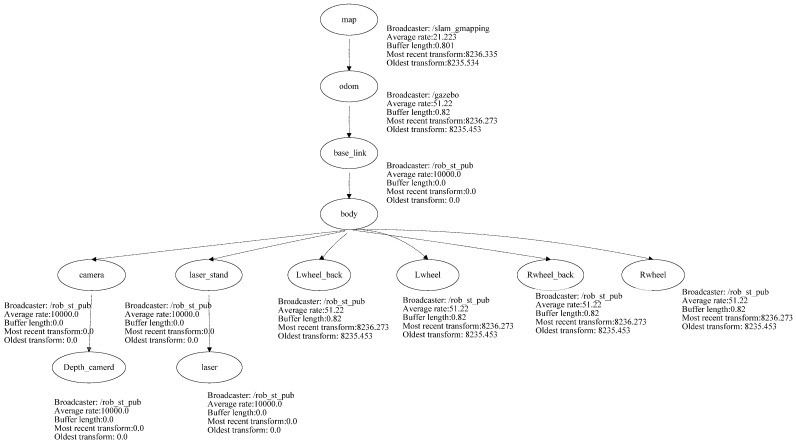
Transform frame tree for the employed rover robot.

The developed robot platform has been simulated into the ROS through the employment of the Gazebo package using the transform frame tree for the simulated robot. The transform tree system keeps track of multiple coordinate frames and maintains the relationship between these coordinates in a tree structure format. In order to simulate the abovementioned robot platform using ROS, this paper used the transform frame tree to convert the physical robot platform into a simulated robot platform in the ROS development environment. All the sensors, actuators, effectors, and controllers have been transformed into the simulation environment. Figure 6 presents the transform frame tree for the employed rover robot platform.

**Figure 7:**
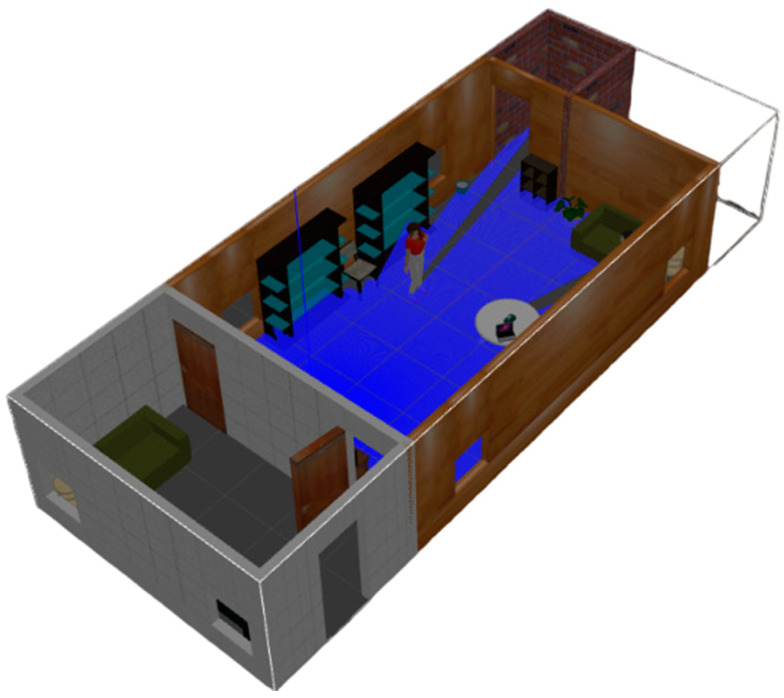
Side view of the experimental indoor testbed area.

**Figure 8:**
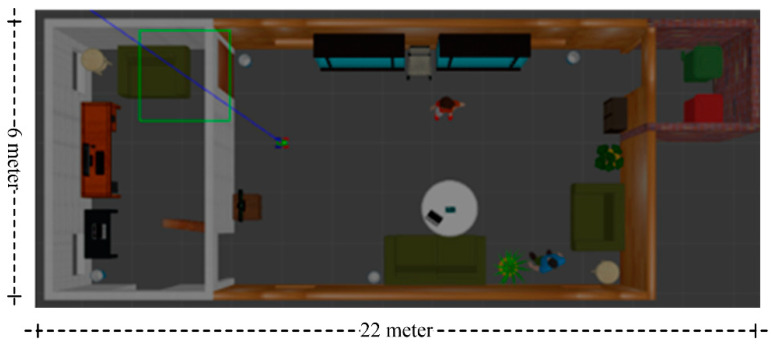
Two-dimensional view of the experimental testbed.

The proposed semantic map production system has been implemented using the ROS environment, simulating an indoor environment with different types of objects. The experiment testbed consists of the following objects: chairs, vases, potted plants, TV monitors, people, trash, a laptop, tissue boxes, tables with breakfast, and large bookshelves. Table 5 presents the objects, along with their total number in the indoor environment, whereas Figure 7 presents a side view of the simulated testbed area including the aforementioned objects. Figure 8 shows a two-dimensional view of the experiment area.

### 4.2. ROS-Based Semantic Map Representation System

**Figure 9:**
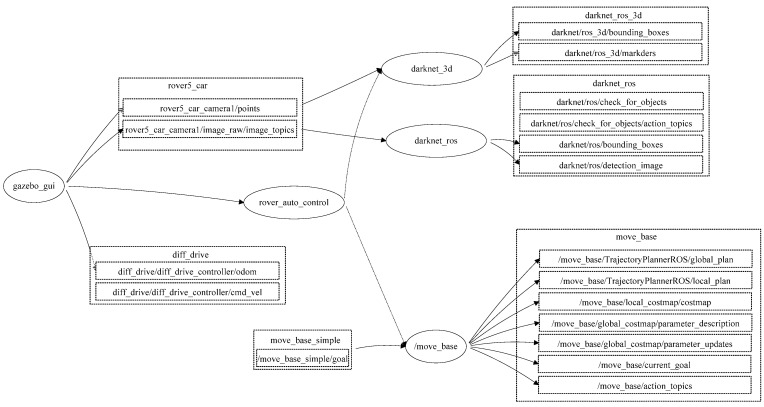
ROS-based architecture for the developed map representation system.

The proposed semantic map production system was implemented using ROS. ROS is a set of tools and libraries that assists in developing reliable robotic applications by providing a structured and modular framework for several robotic tasks [27]. The structure of the developed semantic map representation system using the ROS development environment is presented in Figure 9.

In general, ROS packages are the most basic units of the ROS software, in which each package contains the ROS runtime processes (nodes), libraries, and configuration files. ROS nodes may communicate to each other and exchange data. For the purpose of obtaining an accurate semantic map, this paper developed an ROS package (map_rep package) that consists of several runtime nodes, as presented in Figure 8. The developed system consists of the following main nodes:

1. Gazebogui: This node simulates the developed semantic map representation system on a friendly graphical user interface.
2.    Slam_gmapping: This node builds a 2D map using the LiDAR unit. The data received from the LiDAR unit is used to construct a geometric map, in which the output of this node is a 2D area with geometry information.
3.    Rob_st_pub: This node reveals the current status of the robot platform and broadcasts status information to other nodes for the purpose of exploiting this information in constructing the semantic map area.
4.    Move_base: This node offers an ROS interface for configuring, running, and interacting with the navigation stack on the robot platform. In addition, it controls the robot platform as it moves from one point to another.
5.    N_rvis: This node visualizes the represented map area in 3D, in which the robot platform is visualized using the Rviz package.
6.    Darknet_ros: This is an ROS package for object detection via the employment of the YOLO v3 classification model.
7.    Darknet_ros_3d: This node offers bounding boxes in 3D in order to allow for object distance measurement. Through the employment of an RGB-D camera, the object and its estimated position can be computed.
8.    Rover_auto_control: This node controls other ROS nodes, collecting the necessary LiDAR frames, performing object detection and classification, and finally constructing the semantic map for the area of interest.

The developed nodes communicate with each other using the message function to offer an efficient semantic map. This map includes the geometry information of the area of interest, as well as objects and their 2D locations.

### 4.3. Results

For evaluation purposes, we proposed a set of evaluation metrics that needs to be taken into consideration for the purpose of assessing the efficiency of the developed semantic map production system for robot navigation applications, in which the validation parameters include the following:

1. The recognized objects ratio (*r_o_*): This refers to the total number of objects that have been correctly classified in the area of interest in comparison with the total number of objects in that area. This is expressed as follows:
  $$r_{o}=\sum_{j=0}^{n}\frac{\frac{\sum_{i=0}^{y}k}{m}}{n}$$
  where *j* is the index number of a certain class, *y* is the total number of detected objects in the *kth* class, *m* is the total number of existing objects in the *kth* class, and *n* is the total number of objects in the simulated environment.
2. The object recognition accuracy (*obj_acc_*): This refers to the classification accuracy of recognized objects. Usually, the accuracy is estimated as a percentage of the recognition accuracy. The *obj_acc_* has been estimated using the function presented in YOLO v3 model.
3. The localization error (LE) of detected objects: This measures the average positioning error between the estimated 2D position (*x_e_*, *y_e_*) of an object and its actual 2D position (*x_a_*, *y_a_*) using the Euclidian distance formula, as follows:
  $$LE=\sqrt{{\left(x_{e}-x_{a}\right)}^{2}+{\left(y_{e}-y_{a}\right)}^{2}}$$
4. The geometry map error (*map_err_*): This refers to the error percentage of the geometry map produced by the map production system versus the actual geometry map. It can be estimated using the following formula:
  $$map_{err}=\sqrt{{\left(A_{est}-A_{act}\right)}^{2}}$$
  where *A_est_* is the estimated area of the original map and the *A_act_* is the actual area of the map.
5. The semantic map accuracy: This refers to the difference between the semantic map using the developed system and the actual map area. It can
6. be estimated based on measuring the error of semantic map (*sem_err_*) construction, as follows:
  $${sem}_{err}=\frac{{map}_{err}+r_{o}}{2}$$

First, the total recognized objects parameter is considered. According to the simulation experiments, the ratio of the recognized objects is almost 72.72%, and the robot system was able to recognize almost 16 objects out of 22. For instance, the robot system was able to detect chairs, vases, potted plants, TV monitor, people, and breakfast tables; it failed to detect the laptop located on the desk because it was out of the robot’s view. Table 6 presents the total number of existing and detected objects in the simulation environment.

**Figure 10:**
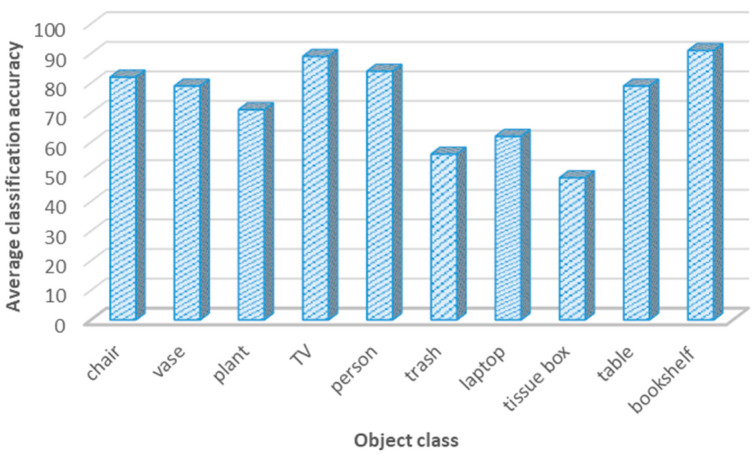
Object classification accuracy for different classes using the YOLO v3 model.

The second parameter was the object classification accuracy. After adopting the YOLO v3 classification model with two different vision datasets, the classification accuracy was around 74.1%; in all cases, the object classification accuracy exceeded the 50%. Figure 10 presents the object classification accuracy for object classes in the simulated environment using the YOLO v3 detection model.

**Figure 11:**
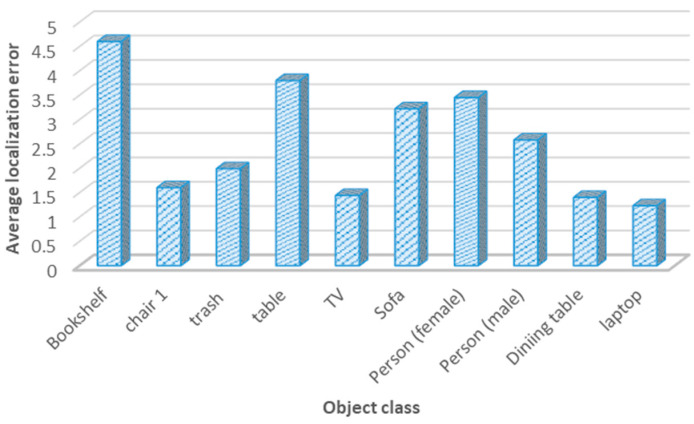
Average localization error for object classes.

The third metric was the localization error for positioning objects in the navigation area. For semantic maps, it is important for the system to place objects correctly to allow the robot to act precisely. Through the adoption of several experimental studies, the average localization error was around 2.67 m. This indicates that the developed system was able to localize the objects of interest with an average localization error of almost 2.8 m, which is considered a reasonable localization error. Figure 11 presents the localization error for 10 categories in the navigation environment.

Moreover, the localization error for object classes is presented in Table 7, which shows the real location of a certain object along with its estimated position by the robot system. This table also displays the Euclidean distance in meters between the real and the measured positions.

**Figure 12:**
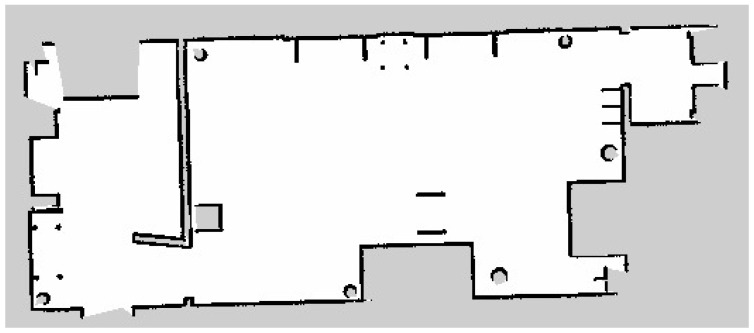
Original geometry map for the indoor environment.

**Figure 13:**
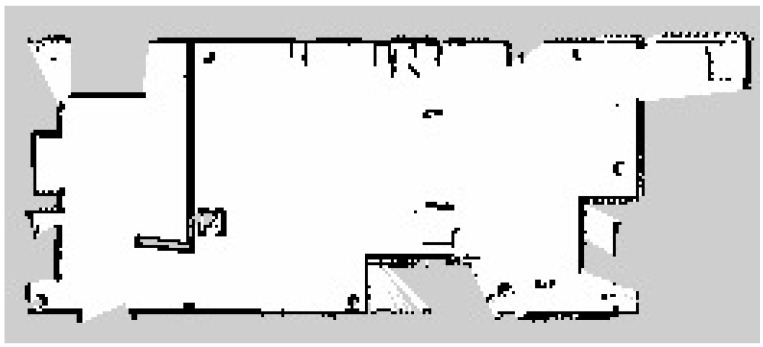
Estimated geometry map for the indoor environment.

The fourth metric was the map production accuracy, which analyzes the efficiency of the developed semantic map production system. The average map production accuracy was almost 85%, with an error of 15%. This indicates that the estimated geometry map is similar to the real map, with only slight errors. Figure 12 presents the original map of the indoor navigation environment. After the robot explored the indoor environment using the LiDAR system, its geometric map is presented in Figure 13.

**Figure 14:**
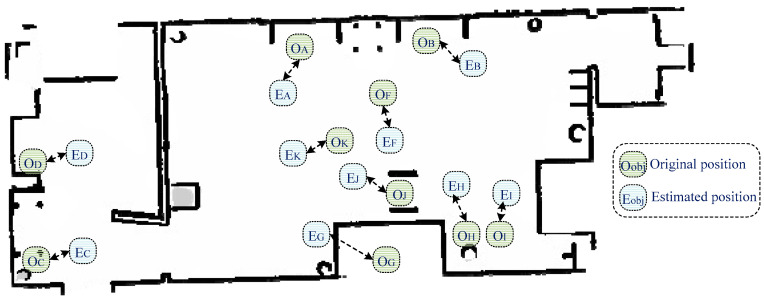
Obtained semantic map, along with the estimated object locations.

Finally, the fifth metric was accuracy of the semantic map. The estimated geometry map was combined with object classification in order to provide an efficient semantic map for robot navigation purposes. Figure 14 presents the obtained semantic map, which contains geometric information and the object’s original and estimated positions. The semantic map construction accuracy was 78.86%, which indicates that the developed system was able to construct an accurate semantic map.

## 5. Discussion

The task of map production is complex because the system must create a geometry map and detect both objects and their locations. Several studies have focused on map production for robot semantic navigation applications, with varying efficiency, reliability, and classification accuracy. For instance, the systems presented in [7,8,9,10] focused on semantic classification using the LiDAR signals with no requirements for vision systems. LiDAR-based classification systems offer reliable classification accuracy with reasonable processing time and cost; however, they offer a limited number of classifications (only one to six classes) due to the nature of data that is collected by the LiDAR unit; this minimizes the accuracy of maps constructed by LiDAR systems.

On the other hand, vision-based systems [11,12,13,15,16] are efficient in terms of the total number of objects that can be detected, as a vision system can capture relevant information about all objects in the area of interest. However, vision-based systems require high processing capabilities in comparison to LiDAR-based systems. In addition, an intensive pre-processing phase is required for training and testing the developed classification model.

Vision-based recognition systems differ in terms of the employed classification model (algorithm); for instance, the work presented in [15,16,18,20] employed a complex classification model, in which high processor capabilities are required to perform the classification task and obtain a reasonable semantic map.

Several object detection and classification models [28,29] with various vision datasets [30,31] have been developed recently. However, it is important to employ an efficient classification model along with a suitable vision dataset in order to easily adopt it with a robot platform and achieve better classification accuracy for robot semantic navigation.

Unlike the recently developed map production systems [21,22], the system proposed in this paper has been validated using reliable evaluation metrics, including the ratio of recognized objects, object recognition accuracy, localization error, geometry map error, and accuracy of the semantic map. Hence, the presented evaluation metrics can be used to assess the efficiency of semantic map production systems.

While the existing research works [11,13,14,17,18] have achieved high classification accuracy with reasonable map production accuracy, these studies did not obtain the geometry map for the navigated environment, and this reduces the efficiency of their semantic maps. Therefore, in this paper, the LiDAR unit was integrated for the purpose of constructing an efficient semantic map that consists of both the geometry information and objects with their corresponding locations on the map.

The developed map production system consists of two main phases; the former involves the production of a geometry map using the LiDAR unit, whereas the latter classifies the objects in the area of interest and determines their locations in the environment. The integration of the results obtained from the prior two phases resulted in a semantic map with rich information. Moreover, the integration of two datasets (COCO and PASCAL) enhanced the classification accuracy and allowed for efficient object detection and recognition accuracy, hence building a semantic map with rich information about the objects in the navigation area, which is a novel strength of the proposed approach.

## 6. Conclusions and Future Work

Robot semantic navigation has received considerable attention recently. This paper proposed and implemented an efficient semantic map production approach that is based on two subsystems, LiDAR and vision, using the ROS development environment through simulating a real-robot platform. In addition, a set of evaluation metrics was presented to assess the efficiency of any semantic map production system. The obtained results proved the efficiency of the developed map production system in terms of object classification, object localization, and semantic map production accuracy. Future work should assess the efficiency of the developed system in several indoor environments with different objects and further enhance the map production accuracy through the development of an accurate classification approach.

## Figures and Tables

**Table 1:** Object classes for Pascal and COCO datasets.

Pascal Dataset	COCO
Person, bird, cat, cow, dog, horse, sheep, airplane, bicycle, boat, bus, car, motorbike, train, bottle, chair, dining table, potted plant, sofa, tv monitor.	Person, bicycle, car, motorbike, airplane, bus, train, truck, boat, traffic light, fire hydrant, stop sign, parking meter, bench, cat, dog, horse, sheep, cow, elephant, bear, zebra, giraffe, backpack, umbrella, handbag, tie, suitcase, frisbee, skis, snowboard, sports ball, kits, baseball bat, baseball glove, skateboard, surfboard, tennis racket, bottle, wine glass, cup, fork, knife, spoon, bowl.

**Table 2:** Summary of the COCO and Pascal datasets.

Dataset	Application	# of Records	# of Classes	Size
COCO	Indoor	330,000	80	25 GB
Pascal	Outdoor	11,530	20	2 GB

**Table 3:** Constructed semantic table.

Ref	Object Id	X-Cord	Y-Cord
1.	12	3.6	7.2
2.	06	8.1	5.4
…	…	…	…

**Table 4:** Specification parameters for the developed robot platform.

Component	Parameter
Robot platform	Rover 2WD
Processor	Raspberry Pi 4 (4 GB RAM)—Raspberry P Ltd, Wales
LiDAR unit	A1 RPLiDAR A1M8
Vision unit	OAK-D Pro—Luxonis
Actuators	2-DC motors—TFK280SC-21138-45
Power source	Lithium battery 11.1 volt 2000 mAh—HRB
Robot speed	10 m/min
Frame per second (FPS)	4
LiDAR frame rate (LFR)	20

**Table 5:** Objects in the Gazebo simulation environment.

Object in Gazebo Simulation	Total
Chair	4
Vase	3
Potted plant	2
TV monitor	1
Person	2
Trash	2
Tissue box	2
Table breakfast	2
Bookshelf large	3

**Table 6:** Total number of classified objects in the simulation environment.

Object in Gazebo Simulation	Exist	Detected	Accuracy
Chair	4	3	75%
Vase	3	2	66%
Potted plant	2	1	50%
TV monitor	1	1	100%
Person	2	2	100%
Trash	2	1	50%
Laptop	1	0	0%
Tissue box	2	1	50%
Table breakfast	2	2	100%
Bookshelf large	3	3	100%

**Table 7:** Localization error for each object in the simulated environment.

Object Class	Tag	Actual (x, y)	Estimated (x, y)	Euclidian Distance (m)
Chair	A	2.723, 1.493	1.476, 0.785	1.43
Bookshelf	B	3.881, 1.662	4.308, −1.861	4.63
Trash	C	−0.707, 1.616	0.837, −0.483	2.60
TV monitor	D	−0.725, −1.265	1.282, 0.673	2.78
Person (female)	F	3.261, 0.636	5.778, −1.737	3.45
Sofa	G	3.054, −2.333	3.125, −0.790	1.54
Plant side	H	4.519, −2.884	3.229, −0.808	2.11
Person (male)	I	5.604, −2.410	2.445, −1.075	3.42
Dining table	J	3.337, 1.904	1.290, 1.443	2.86
Laptop	K	2.846, 1.509	1.094, 0.567	1.98
Average localization error				2.67

## Data Availability

This study employed two datasets to perform semantic classification, as follow: First, the MS-COCO vision dataset, where it can be accessed through: https://cocodataset.org/#home (accessed on 7 May 2024), and Second, the PASCAl dataset that can be accessed through: http://host.robots.ox.ac.uk/pascal/VOC/ (accessed on 10 May 2024).

## References

[1] Gul F., Rahiman W., Alhady S.S.N. (2019). A comprehensive study for robot navigation techniques. Cogent Eng..

[2] Alamri S., Alamri H., Alshehri W., Alshehri S., Alaklabi A., Alhmiedat T. (2023). An Autonomous Maze-Solving Robotic System Based on an Enhanced Wall-Follower Approach. Machines.

[3] Alhmiedat T. (2023). Fingerprint-Based Localization Approach for WSN Using Machine Learning Models. Appl. Sci..

[4] Alamri S., Alshehri S., Alshehri W., Alamri H., Alaklabi A., Alhmiedat T. (2021). Autonomous Maze Solving Robotics: Algorithms and Systems. Int. J. Mech. Eng. Robot. Res..

[5] Crespo J., Castillo J.C., Mozos O.M., Barber R. (2020). Semantic Information for Robot Navigation: A Survey. Appl. Sci..

[6] Alqobali R., Alshmrani M., Alnasser R., Rashidi A., Alhmiedat T., Alia O.M. (2023). A Survey on Robot Semantic Navigation Systems for Indoor Environments. Appl. Sci..

[7] Alenzi Z., Alenzi E., Alqasir M., Alruwaili M., Alhmiedat T., Alia O.M. (2022). A Semantic Classification Approach for Indoor Robot Navigation. Electron..

[8] García F., Jiménez F., Naranjo J.E., Zato J.G., Aparicio F., Armingol J.M., de la Escalera A. (2011). Environment perception based on LIDAR sensors for real road applications. Robotica.

[9] Álvarez-Aparicio C., Guerrero-Higueras .M., Rodríguez-Lera F.J., Clavero J.G., Rico F.M., Matellán V. (2019). People Detection and Tracking Using LIDAR Sensors. Robotica.

[10] Von Haaren C., Warren-Kretzschmar B., Milos C., Werthmann C. (2014). Opportunities for design approaches in landscape planning. Landsc. Urban Plan..

[11] Ma F., Cavalheiro G.V., Karaman S. Self-Supervised Sparse–to–Dense: Self-Supervised Depth Completion from LiDAR and Monocular Camera. Proceedings of the International Conference on Robotics and Automation (ICRA).

[12] Bruno D.R., Osorio F.S. A Comparison of Traffic Signs Detection Methods in 2D and 3D Images for the Benefit of the Navigation of Autonomous Vehicles. Proceedings of the Latin American Robotic Symposium, Brazilian Symposium on Robotics (SBR) and 2018 Workshop on Robotics in Education (WRE).

[13] Dang T.-V., Bui N.-T. (2023). Multi-Scale Fully Convolutional Network-Based Semantic Segmentation for Mobile Robot Navigation. Electronics..

[14] Deng W., Huang K., Chen X., Zhou Z., Shi C., Guo R., Zhang H. (2020). Semantic RGB-D SLAM for Rescue Robot Navigation. IEEE Access.

[15] Teso-Fz-Betoño D., Zulueta E., Sánchez-Chica A., Fernandez-Gamiz U., Saenz-Aguirre A. (2020). Semantic Segmentation to Develop an Indoor Navigation System for an Autonomous Mobile Robot. Mathematics.

[16] Ferri G., Caselli E., Mattoli V., Mondini A., Mazzolai B., Dario P. (2008). SPIRAL: A novel biologically-inspired algorithm for gas/odor source localization in an indoor environment with no strong airflow. Robot. Auton. Syst..

[17] Fang B., Mei G., Yuan X., Wang L., Wang Z., Wang J. (2021). Visual SLAM for robot navigation in healthcare facility. Pattern Recognit..

[18] Honda A., James S. Averaging aggregation functions based on inclusion-exclusion integrals. Proceedings of the 2017 Joint 17th World Congress of International Fuzzy Systems Association and 9th International Conference on Soft Computing and Intelligent Systems (IFSA-SCIS).

[19] Barfield W. (2018). Liability for Autonomous and Artificially Intelligent Robots. Paladyn, J. Behav. Robot..

[20] Qi X., Wang W., Liao Z., Zhang X., Yang D., Wei R. (2020). Object Semantic Grid Mapping with 2D LiDAR and RGB-D Camera for Domestic Robot Navigation. Appl. Sci..

[21] Zheng C., Du Y., Xiao J., Sun T., Wang Z., Eynard B., Zhang Y. (2025). Semantic map construction approach for hu-man-robot collaborative manufacturing. Robot. Comput. Integr. Manuf..

[22] Zhao C., Mei W., Pan W. (2015). Building a grid-semantic map for the navigation of service robots through human–robot interaction. Digit. Commun. Netw..

[23] Dos Reis D.H., Welfer D., Cuadros M.A.D.S.L., Gamarra D.F.T. (2019). Mobile Robot Navigation Using an Object Recognition Software with RGBD Images and the YOLO Algorithm. Appl. Artif. Intell..

[24] Henke dos Reis D., Welfer D., de Souza Leite Cuadros M.A., Tello Gamarra D.F. (2019). Object Recognition Software Using RGBD Kinect Images and the YOLO Algorithm for Mobile Robot Navigation. Intelligent Systems Design and Applica-tions: 19th International Conference on Intelligent Systems Design and Applications.

[25] Chehri A., Zarai A., Zimmermann A., Saadane R. 2D autonomous robot localization using fast SLAM 2.0 and YOLO in long corridors. Proceedings of the International Conference on Human-Centered Intelligent Systems.

[26] Alotaibi A., Alatawi H., Binnouh A., Duwayriat L., Alhmiedat T., Alia O.M. (2024). Deep Learning-Based Vision Systems for Robot Semantic Navigation: An Experimental Study. Technologies.

[27] Alhmiedat T., Marei A.M., Messoudi W., Albelwi S., Bushnag A., Bassfar Z., Alnajjar F., Elfaki A.O. (2023). A SLAM-Based Localization and Navigation System for Social Robots: The Pepper Robot Case. Machines.

[28] Zaidi S.S.A., Ansari M.S., Aslam A., Kanwal N., Asghar M., Lee B. (2022). A survey of modern deep learning based object detection models. Digit. Signal Process..

[29] Dhillon A., Verma G.K. (2019). Convolutional neural network: A review of models, methodologies and applications to object detection. Prog. Artif. Intell..

[30] Kuznetsova A., Rom H., Alldrin N., Uijlings J., Krasin I., Pont-Tuset J., Kamali S., Popov S., Malloci M., Kolesnikov A. (2020). The Open Images Dataset V4. Int. J. Comput. Vis..

[31] Bayoudh K., Knani R., Hamdaoui F., Mtibaa A. (2021). A survey on deep multimodal learning for computer vision: Advances, trends, applications, and datasets. Vis. Comput..

